# Sphenopalatine Ganglion Acupuncture Improves Nasal Ventilation and Modulates Autonomic Nervous Activity in Healthy Volunteers: A Randomized Controlled Study

**DOI:** 10.1038/srep29947

**Published:** 2016-07-18

**Authors:** Kuiji Wang, Luquan Chen, Yang Wang, Chengshuo Wang, Luo Zhang

**Affiliations:** 1 Department of Otolaryngology Head and Neck Surgery, Beijing TongRen Hospital, Capital Medical University, Beijing 100730, China; 2 Beijing Key Laboratory of Nasal Diseases, Beijing Institute of Otolaryngology, Beijing 100005, China; 3 Department of Traditional Medicine, Beijing TongRen Hospital, Capital Medical University, Beijing 100730, China

## Abstract

The study aimed to assess the effects of Sphenopalatine ganglion (SPG) acupuncture on nasal ventilation function and autonomic nervous system in health volunteers. 39 healthy subjects were randomly assigned to either active SPG acupuncture group (AA group) or sham-SPG acupuncture group (SA group). All subjects were assessed for self-reported nasal ventilation, nasal patency (nasal airway resistance (NAR) and nasal cavity volume (NCV), exhaled nasal nitric oxide (nNO), and neuropeptides (substance P(SP), vasoactive intestinal peptide (VIP) and neuropeptide Y (NPY)) in nasal secretions at baseline, 30 minutes, 2 hours, and 24 hours after acupuncture. Significantly more subjects in AA group reported improvements in nasal ventilation at all time points after acupuncture, compared to SA group. NAR and NCV were also significantly lower in AA group than SA group. The level of nNO in AA group was significantly decreased after 24 hours compared to SA group. The level of NPY was significantly increased in AA group at 30 minutes and 2 hours compared to baseline and SA group. The levels of SP and VIP were not significantly different in the two groups. We concluded that SPG acupuncture could help to improve nasal ventilation by increasing sympathetic nerve excitability in healthy volunteers.

Acupuncture is a traditional form of Chinese medicine, which can be traced back to 2500 years ago, and more recently has been widely used as a therapeutic modality for various otolaryngology disorders^1^,^2^. A practitioner survey has indicated that about 99% of licensed acupuncturists treating patients for chronic nasal and sinus symptoms perceived acupuncture treatment as being highly efficacious^3^. Indeed, several recent randomized controlled trials have confirmed the efficacy of acupuncture in the treatment on nasal inflammatory disease, including allergic rhinitis, and vasomotor rhinitis^4^,^5^,^6^,^7^,^8^,^9^,^10^,^11^,^12^,^13^. Furthermore, the latest American Clinical Practice Guideline for Allergic Rhinitis recommends that acupuncture may be offered as an option for patients with an interest in non-pharmacologic approaches to management of allergic rhinitis^14^.

In conventional acupuncture for nasal inflammatory disease, needles are inserted at specific acupoints (termed Xuewei in Chinese) in the body and gently rotated until the patient feels “de-qi sensation” (i.e. a subjective feeling of severe electric shock at the ipsilateral buccal region or a feeling of water splashing in the nose). Xuewei is pure concept of traditional Chinese medicine, which is difficult to explain by modern medicine, and therefore difficult to standardize. Indeed, different manoeuvres performed by the acupuncturist at the same Xuewei could result in different efficacy. However, two recent studies have indicated that stimulation of sphenopalatine ganglion (SPG) by acupuncture can improve nasal symptoms and quality of life in nasal inflammatory diseases^10^,^15^; thus suggesting that acupuncture-stimulated neural regulation may offer an alternative form of therapy for management of nasal inflammatory disease.

Neural mechanisms play an important role in the pathophysiology of nasal inflammatory diseases; with there being complex interplay between nasal mucosa inflammation and neural effects^16^. The normal nasal physiological function depends upon a precise regulation of the autonomic nervous system (ANS), including the nociceptive, parasympathetic and sympathetic nerves. It is recognized that SPG plays a critical and delicate coordinating role in the regulation of vascular, glandular and other processes^17^. The SPG is located in the pterygopalatine fossa, which is composed of sympathetic, parasympathetic and sensory nerves in nasal cavity. Parasympathetic activation would lead to nasal glandular secretion. VIP is an appropriate marker of parasympathetic activation. On the other hand, sympathetic nerves pass through the sphenopalatine ganglia via the vidian canal and contain colocalized NPY, which causes a slightly delayed but prolonged vasoconstriction effect. Vasoconstriction decreases mucosal vascular blood volumes with collapse of sinusoids and thinning of the nasal mucosa^18^. Thus, the anticipated effect would be increased nasal cavity volume and decreased nasal airflow resistance.

We hypothesised that the effect of SPG acupuncture treatment might be correlated with modulating the imbalance between the parasympathetic and sympathetic activity in the nose. The aim of the present study was therefore to evaluate the effects of SPG acupuncture on nasal ventilation/patency and the modulation of ANS activity.

## Methods

### Study Subjects and study design

The study was a single-centre, randomized, sham acupuncture-controlled, patient assessor-blinded trial, conducted in Beijing TongRen Hospital from 12^th^ March 2014 to 24^th^ March 2015. All volunteers were college freshman who majored in Western Medicine recruited from the Capital Medical University. The subjects had little or no knowledge of Chinese traditional medicine and none had received any previous acupuncture therapy; thus, being unaware of the anticipated de-qi sensory experience. All subjects were healthy non-smoking volunteers, and to be eligible for enrolment to the study, were required to have no history of nasal disease (allergic rhinitis, nasal polyps, rhinosinusitis, nasal septum deviation and history of nose surgery) and not have suffered from respiratory tract infections for 4 weeks before the study. All participants were additionally required to exhibit negative skin prick test (SPT) reactions to common allergens at screening.

**Figure 1:**
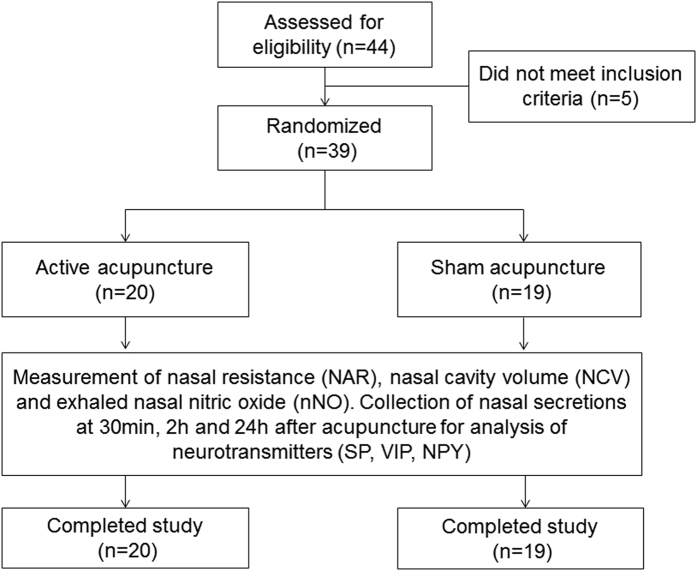
Study flow-chart.

Eligible subjects were randomly assigned to either the active SPG acupuncture group (AA group) or the sham-SPG acupuncture group (SA group) according to a computer-generated random allocation sequence, by an experienced acupuncture practitioner who was provided with the randomization sequence in a sealed envelope. Following acupuncture, the patients were assessed for self-reported nasal symptoms, nasal airway resistance, nasal cavity volume and exhaled nNO, at baseline, and 30 min, 2 h and 24 h after acupuncture. Nasal secretions were also collected at these time points for analysis of neuropeptides (Fig. 1). All subjects and investigators involved in the study, as well as the statistician analysing the data were blinded to the treatment groups.

The study protocol was approved by the Ethics Committee of Beijing Institute of Otolaryngology, and a written informed consent was obtained from each participant before the study began. The experiment was performed in accordance with approved guidelines. The trial was registered on the public database ClinicalTrials.gov in November 2015 (NCT02603588).

### Interventions

All acupuncture treatments were performed by an acupuncture practitioner with more than 8 years of clinical acupuncture experience; using disposable needles 60 mm in length and 0.35 mm in diameter (Dongbang Medical Apparatus Co.Ltd.,Suzhou, China). In the case of subjects in the AA group, the acupuncture point was selected in the sphenopalatine ganglion (unilateral side). The acupuncture needle was inserted from the lower border of the zygomatic arch, slightly posterior to the suture protuberance between the zygomatic process and temporal process and about 1.5 cm anterior to the Xiaguan (St 7) point, which was felt as an arch depression. The needle was inserted at the middle of the arch depression, and avoiding the bone tissue, the needle was directed obliquely anteriorly, until nearly the whole needle was beneath the skin. The needle was rotated until the participant felt “de-qi” sensations; and then twirled, lifted and thrust 3–5 times to give repeated stimulation. The process of the acupuncture took about 1 minute and needed about 2 minutes pressing on the skin to prevent bleeding. In the case of subjects in the SA group, the needle was inserted at the selected acupuncture site to a depth of only 2–3 cm, and the procedure of rotating, twirling and thrusting the needle was repeated, in order to blind the subject to the sham treatment. As the sphenopalatine ganglion is located in a deeper position at over 50 mm from the point of acupuncture, the sham-acupuncture resulted in some pain, but not the “de-qi” sensation.

**Figure 2:**
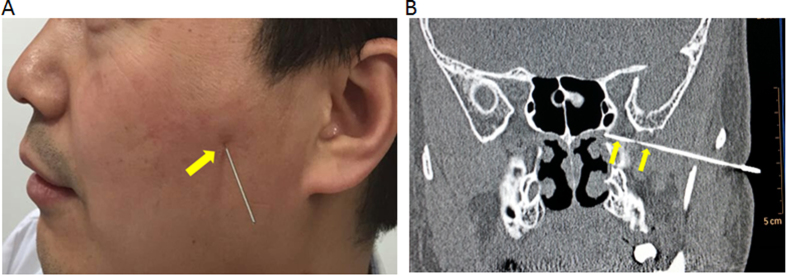
(**A**) Site of acupuncture in sphenopalatine ganglion(yellow arrow); (**B**) High resolution CT scan of pterygopalatine fossa in coronal plane after the acupuncture showing needle penetration intopterygopalatine fossa(yellow arrows).

During the acupuncture process, the acupuncturist sat on the side of the participant and in a position where the participant could not see the acupuncturist’s face or the length of the needle. Throughout the procedure the acupuncturist monitored the subjects’ responses closely and avoided repeatedly asking the participant if they felt any pain. In this regard, when the participant reported sudden feeling of severe electric shock at the ipsilateral buccal region or feeling of water splashing in the nose, acupuncture was considered to be successful. In order to confirm that the selected acupuncture point was accurate and that the needle had penetrated the sphenopalatine ganglion, high resolution sinus CT scans were performed on a pilot group of five participants prior to conducting the full study (Fig. 2).

The main outcomes were the changes of self-reported nasal ventilation, objective nasal patency measurements, exhaled nasal nitric oxide and the change in neuropeptides in nasal secretions; all assessed four times in a time-dependent manner at baseline, and then at 30 minutes, 2 hours, and 24 hours after acupuncture.

### Self-reported nasal ventilation

One nominated investigator asked all participants the question “How does your nasal ventilation feel?” at each time point following acupuncture, and the subjective perception of nasal ventilation was recorded by each participant according to one of three categories: unchanged, better, and worse.

### Nasal patency

Eccovision acoustic rhinometry (Hood Labs, Pembroke, USA) was used to measure the total nasal cavity volume (NCV) and the ATMO 300 Rhinomanometer (ATMOS MedizinTechnikGmbH&Co., Feldkirch, Germany) was used to measure the nasal airway resistance (NAR) at 75 Pa point (R_75T_) by anterior active rhinomanometry. The total nasal airway resistance was calculated using the formula (R_t_ = R_l_·R_r_/R_l_ + R_r_). All measurements were performed at a temperature of 24 ± 1 °C and 70% ± 1% humidity.

### Exhaled nNO

Exhaled nNO was measured using a nitric oxide analyser (Niox; Aereocrine, Solna, Sweden). NO-free air was aspirated at a flow rate of 50 ml/s through the nasal cavity and the subject was asked to exhale against the air-resistance, resulting in an intraoral pressure of approximately 10 cm H_2_O in order to close the velum. Nasal gas was continuously routed into the analyser and nNO was measured from a plateau lasting for at least 3 s. The procedure was repeated three times and a mean concentration of nNO (parts per billion, ppb) was calculated.

### Side effects of acupuncture

Any side effects following acupuncture were recorded. The intensity of pain during acupuncture was assessed using a visual analogue scale (VAS) of 0–10 cm; where 0 = minimum pain intensity and 10 = maximum pain intensity.

### Neuropeptides in nasal secretions

Nasal secretions were collected and processed as described previously^19^. Briefly, nasal secretions were obtained by inserting a postoperative sinus sponge pack in the nasal cavity (acupuncture side) for five minutes. The quantity of secretions collected in each sponge was determined by comparing the weight of the sponge before and after insertion into the nasal cavity, and then 2 mL of 0.9% sodium chloride solution was added to the sponge. All sponges were stored at 4 °C for two hours and then transferred to a 5 mL BD syringe. The bulk of the nasal secretions was forced out of the sponges using the piston of the syringe, and centrifuged at 1500 g for 15 minutes at 4 °C. The supernatants were separated and stored in aliquots at −20 °C; until analysis for the levels of neuropeptides including substance P, VIP and NPY using commercial ELISA kits (R&D Systems, Minneapolis, Minn).

### Statistical analyses

Data analyses were performed using the SPSS statistical package (version 19.0, SPSS Inc., Chicago, IL). The differences in NAR, NCV, nNO, SP, VIP, and NPY were compared by analysis of variance (ANOVA) for repeated measures between the two groups. Comparisons within groups between baseline and after acupuncture data were performed by multiple comparisons in ANOVA. The sex ratio and the change of nasal ventilation were compared using Chi-squared tests. The age and the intensity of pain were calculated and compared between the groups by independent-samples *t* test. A value of *P* < 0.05 (two-tailed) was considered to be statistically significant.

### Trial Registration

Clinicaltrials.gov NCT02603588. The clinical trials registered with ClinicalTrial.gov under the title “Effects of Sphenopalatine Ganglion Acupuncture on Nasal Ventilation and Autonomic Nervous Activity in Healthy Volunteers”.

## Results

### Demographic data

Overall, 44 participants were screened. Among the participants, 5 participants did not meet the inclusion criteria, and thus 39 subjects were randomised to either the AA group (n = 20) or the SA group (n = 19). All 39 participants completed the trial. The two study groups were not significantly different with respect to demographic and clinical characteristics (Table 1).

### Outcome measures

#### Self-reported nasal ventilation

Significantly greater number of subjects in the AA group (80–95%) reported nasal ventilation to be better at 30 minutes, 2 hours and 24 hours after acupuncture, compared to subjects in the SA group. In contrast, the majority of subjects in the SA group reported nasal ventilation to be unchanged at any time-point after acupuncture (Table 2).

#### Side effects of acupuncture

**Figure 3:**
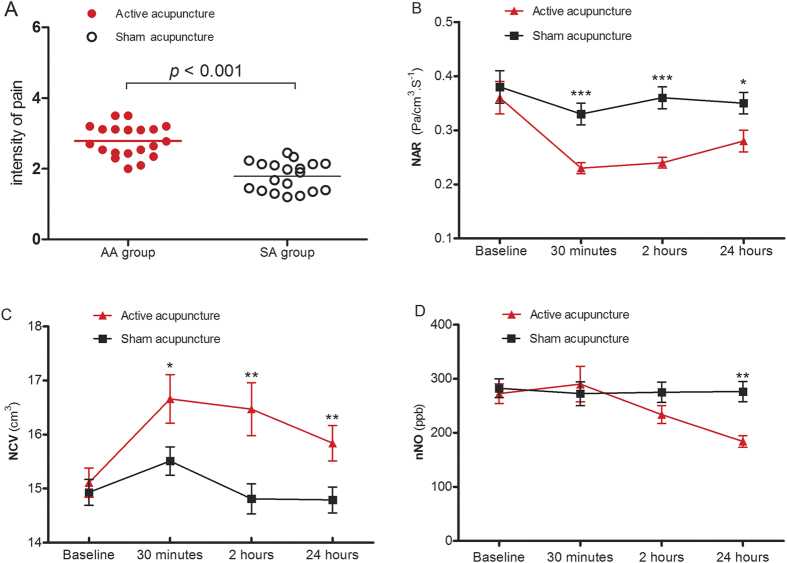
The change in intensity of pain, nasal patency and exhaled nasal nitric oxide, following acupuncture. (**A**) The intensity of the pain induced by acupuncture; (**B**) NAR: Total nasal airway resistance at 75 Pa (Pa·cm^−3^·s^−1^); (**C**) NCV: Total nasal cavity volume from 0–5 cm from the nostril (cm^3^); (**D**) nNO: exhaled nasal nitric oxide (ppb). Data are presented as mean ± SEM values. The differences in NAR, NCV and nNO were compared by analysis of variance (ANOVA) for repeated measures between the two groups. The intensity of pain was calculated and compared between the groups by independent-samples *t* test. *P < 0.05, **P < 0.01, ***P < 0.001.

All subjects tolerated the acupuncture procedure well. However, subjects in both groups reported acupuncture-induced pain, which was reported to be significantly more intense in the AA group than in the SA group, as assessed by VAS (2.79 ± 0 .44 *vs* 1.78 ± 0.41, *P* < 0.001) (Fig. 3A). In the AA group 15/20 participants complained of sharp pain, and 5/20 participants complained of dull pain. Similarly, in the SA group 16/19 participants complained of dull pain and 3/19 participants complained of sharp pain; however, the degree of pain was significantly less than in the AA group. Overall, only 1/39 participant complained of steady pain, and none complained of throbbing, burning, other subjective pain interpretations, itch or cold sensations due to acupuncture.

#### Nasal patency

Assessment of nasal patency following acupuncture demonstrated that NAR in the AA group was significantly improved from baseline levels at 30 minutes (0.23 ± 0.01 *vs.* 0.36 ± 0.03, *P* < 0.001), 2 hours (0.24 ± 0.01 *vs*. 0.36 ± 0.03, *P < *0.001), and 24 hours (0.28 ± 0.02 *vs*. 0.36 ± 0.03, *P* = 0.012; whereas significant improvement in NAR in the SA group was noted only after 30 minutes (0.33 ± 0.02 *vs*. 0.38 ± 0.03, *P* = 0.044) (Table 3). Comparison of the change in NAR between the two treatment groups further demonstrated that NAR was significantly lower in the AA group than the SA group at 30 minutes (0.23 ± 0.01 *vs*. 0.33 ± 0.02, *P* < 0.001), 2 hours (0.24 ± 0.01 *vs*. 0.36 ± 0.02, *P* < 0.001) and 24 hours (0.28 ± 0.02 *vs*. 0.35 ± 0.02, *P* = 0.027) (Table 3 and Fig. 3B). Similarly, NCV was significantly higher in the AA group than in the SA group at all time-points investigated following acupuncture (Table 3 and Fig. 3B,C).

#### Exhaled nasal nitric oxide

The concentration of nNO was not significantly altered from baseline in either treatment group at 30 minutes or 2 hours following acupuncture, however, the level of nNO in the AA group was significantly decreased at 24 hours after acupuncture compared to SA group (184.0 ± 10.98 *vs*. 276.4 ± 18.82, *P* < 0.001) (Fig. 3D).

#### Neuropeptides

**Figure 4:**
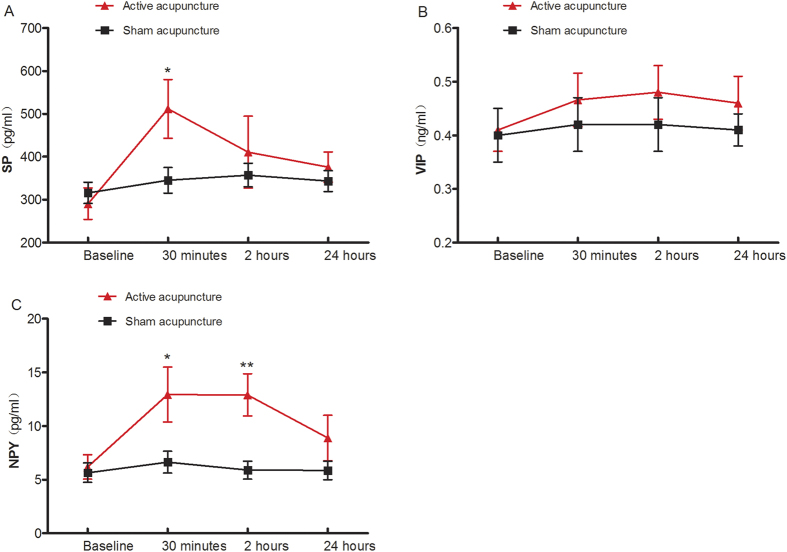
The change in the level of neuropeptides in nasal secretion, following acupuncture. (**A**) SP: substance P (pg/ml); (**B**) VIP: vasoactive intestinal peptide (ng/ml); (**C**) NPY: neuropeptide Y (pg/ml). Data are presented as mean ± SEM values. The differences of SP, VIP and NPY were compared by analysis of variance (ANOVA) for repeated measures between the two groups. *P < 0.05, **P < 0.01.

Active acupuncture significantly increased the levels of SP at 30 mins compared to baseline (511.5 ± 68.24 *vs.* 290.5 ± 36.89, *P* = 0.011) and sham acupuncture (511.5 ± 68.24 *vs.* 345.1 ± 29.89, *P* = 0.036), although the levels of SP were not different at 2 hours and 24 hours. The level of SP was not significantly altered from baseline at any time point in the SA group. Similarly, assessment of the level of VIP indicated that this neuropeptide was not significantly altered at any time point following either the active or sham acupuncture treatment; whereas the level of NPY in the AA group was significantly increased at 30 minutes and 2 hours compared to the baseline (12.94 ± 2.56 *vs.* 6.19 ± 1.13, *P* = 0.021; 12.91 ± 1.96 *vs.* 6.19 ± 1.13, *P* = 0.022, respectively) and compared tothe SA group (12.94 ± 2.56 *vs.* 6.64 ± 1.02, *P* = 0.034; 12.91 ± 1.96 *vs.* 5.90 ± 0.84, *P* = 0.003, respectively). In contrast, the level of NPY in the SA group was increased significantly at only 30 minutes after treatment compared to baseline (6.64 ± 1.02 *vs.* 5.65 ± 0.91, *P* = 0.011)(Table 3 and Fig. 4A–C).

## Discussion

To our knowledge, this is the first randomized, controlled study to investigate the effect of SPG acupuncture on nasal ventilation and patency and suggests that SPG acupuncture may significantly improve self-reported nasal ventilation and nasal patency, as indicated by objective measurements of NAR and NCV, in healthy volunteers. Furthermore, SPG acupuncture may significantly decrease the levels of exhaled nNO and increase the level of NPY; but not levels of VIP and SP in nasal secretions, of these individuals. Moreover, SPG acupuncture appears to be a relatively safe and a well-tolerated treatment.

Nasal congestion is one of the most common nasal symptoms suffered by patients referred to a rhinology clinic. Although many patients choose frequent treatment with topical decongestants for symptomatic relief of nasal congestion, long-term application of decongestants lead to rebound exacerbation of nasal congestion and detriment of the nasal mucosa. In this respect, evidence from two recent prospective, randomized, controlled trials has suggested that acupuncture at specific points related to nasal congestion may offer a suitable alternative treatment option, as this significantly improved nasal congestion in subjects with hypertrophic inferior turbinate or chronic sinusitis without polyps, compared with sham acupuncture^8^,^9^. The finding from the present study is in accordance with these findings; however, unlike these studies which measured the effect at 20–30 minutes after acupuncture, our study has additionally demonstrated that the effect of SPG acupuncture was evident even at 24 hours after acupuncture. Our finding that NAR was also significantly decreased in the sham acupuncture group only at 30 minutes compared to baseline suggests that this may possibly be a consequence of the probe causing pain, which resulted in sympathetic responses with nasal vasoconstriction.

It is well known that NO plays a role in host defence, ciliary activity, and particularly inflammation in the airways^20^, which has therefore led to the measurement of NO in exhaled nasal air as an indirect marker of nasal inflammation^21^,^22^. The present study demonstrated that SPG acupuncture significantly decreased the level of nNO in healthy volunteers compared to sham acupuncture. This finding is in accordance with other studies. Lee and colleagues^23^ have shown that carrageenan-induced expression of inducible nitric oxide synthase, which is known to generate nitric oxide, was inhibited by 2 Hz electro-acupuncture treatment in animals. It has also been shown that acupuncture treatment can progressively decrease the levels of serum NO in migraine patients^24^. As NO has been shown to be involved in vasodilatation, the finding for a decrease in nNO levels in the present study suggests that that SPG acupuncture may partly lead to decreased nasal congestion by attenuating NO-induced vasodilation and subsequent swelling of the nasal mucosa. However, this hypothesis needs to be confirmed in further studies investigating patients with nasal inflammatory disease. It is interesting that in the current study there was also a decrease in nNO at 24 hours after acupuncture in AA group. One possible explanation is that rebound vasodilation following neurogenic vasodilation after probe injury might have caused mucosa swelling that obstructed the maxillary sinus; a major source of nasal NO. Again this hypothesis needs to be confirmed in further studies.

SPG has a close association with multiple nerves innervating the nasal cavity and has been known as the first relay station of the autonomic fibres; suggesting that SPG may serve as a therapeutic target in autonomic imbalance situations. Evidence indicates that dysfunction of the ANS may be particularly relevant to the pathogenesis of nasal inflammatory diseases^16^,^25^,^26^. Extensive research has shown that sympathetic hypofunction is important in allergic rhinitis and vasomotor rhinitis. Kaliner and colleagues^27^ found that patients with allergic rhinitis showed abnormal adrenergic hyporeactivity and cholinergic hyperreactivity, which may contribute to the pathogenesis of allergic diseases. Similarly, another study assessed the relationship between allergic rhinitis and ANS dysfunction, and suggested that the hypoactive sympathetic nervous system was an important feature of allergic rhinitis, as indicated by significantly abnormal ANS scores in allergic rhinitis patients^28^. Additionally, it has been suggested that the dysfunction in ANS disorders, characterized by a hypoactive sympathetic nervous system, was one of important causes of vasomotor rhinitis^29^,^30^.

The mechanisms underlying the efficacy of acupuncture are not clearly understood. A review by Takahashi^31^ has suggested that acupuncture may regulate the imbalance between the parasympathetic and sympathetic activity. More recently, studies have examined the mechanisms underlying the anti-inflammatory effects of acupuncture in allergic rhinitis, which facilitated modulation of the nervous, endocrine and immune systems^32^,^33^. Overall, these studies indicated that the anti-inflammatory mechanisms of acupuncture appear to be mediated by down-regulation of pro-inflammatory neuropeptide, neurotrophins, Th2 cytokines and pro-inflammatory cytokines, thus enhancing the Th1/Th2 balance towards Th1 direction.

Traditional acupuncture works by stimulating the Xuewei (specific acupoints), which do not easily explain the efficacy and mechanism according to modern medicine theory. In contrast, it is not difficult to postulate the mechanisms underlying SPG acupuncture, as stimulation of the sphenopalatine ganglion will directly lead to stimulation of the nerve branches, resulting in corresponding neurological effects. Thus, SPG acupuncture can be considered as a novel form of needle probing into a site or region that is not a traditional Chinese acupuncture site, and should not be interpreted as a rationalization of acupuncture.

Neuropeptides play an important role in the pathogenesis of allergic rhinitis; leading to vasodilation and vascular leakage, and subsequent nasal blockage and rhinorrhoea^34^. In the current study, the levels of neuropeptides including substance P, VIP and NPY; which have been shown to be involved in allergic rhinitis^34^,^35^; were evaluated in nasal secretions of healthy volunteers to assess the effect of acupuncture on the ANS. Substance P has been shown to be present in type C nociceptive sensory neurons of the human nasal mucosa and found to be both involved in the late phase of allergic reaction and to induce histamine release from nasal mucosa in allergic rhinitis patients^36^. VIP, released by parasympathetic reflexes, has important functions in nasal cavity, such as glandular discharge and vasodilatation. Similarly, its receptor has been shown to be increased in the nasal mucosa of allergic rhinitis^37^,^38^. In the present study, the level of SP was significantly increased in the SPG acupuncture group compared to sham acupuncture group only at 30 minutes after acupuncture, which appears to be consistent with the intensity of pain induced by the direct stimulation of the subset of sensory nerves in SPG. The finding that VIP levels were not significantly altered before or after acupuncture in these subjects, suggests that SPG acupuncture might not have any or a particularly strong stimulatory effect on the parasympathetic nerves. In contrast, our finding that NPY, a potent vasoconstrictor peptide found in sympathetic neurons and which regulates vasomotor tone in the human nasal mucosa^39^, was significantly increased in the nasal secretionsby 30 minutes and 2 hours after SPG acupuncture compared to sham acupuncture, suggests that sympathetic nerve stimulation might play a critical role in SPG acupuncture.

In conclusion, the present study has demonstrated that SPG acupuncture leads to significant improvement in nasal ventilation and nasal patency, and increased sympathetic nerve excitability in health volunteers. Although the SPG is not a traditional Chinese acupuncture site, and the findings from the current study cannot therefore be directly compared with the traditional medical acupuncture techniques, these findings nevertheless suggest that deep probing of the SPG might offer a novel effective and safe treatment strategy for controlling the symptoms of nasal inflammatory diseases. Despite these positive findings, the present study is somewhat limited in that participants were healthy volunteers and may not be representative of patients with nasal inflammatory diseases. Furthermore, the sample size was relatively small and the study period short, which did not allow assessment of a long-term effect of acupuncture. It is possible that insertion of a metallic probe into the electrically conducive SPG could cause tissue disruption, neuronal depolarization, and neurotransmitter release at secretory varicosities in the end organs. In this regard, use a plastic probe in future studies would adequately control for these adverse effects of a metallic probe in the acupuncture of SPG. Thus, these findings need to be confirmed in further trials involving larger groups of patients with inflammatory nasal disease over longer time spans, and in consideration of the limitations of this study.

## Additional Information

**How to cite this article**: Wang, K. *et al*. Sphenopalatine Ganglion Acupuncture Improves Nasal Ventilation and Modulates Autonomic Nervous Activity in Healthy Volunteers: A Randomized Controlled Study. *Sci. Rep.* **6**, 29947; doi: 10.1038/srep29947 (2016).

## Figures and Tables

**Table 1:** Baseline characteristics of subjects.

Characteristic	AA group (n = 20)	SA group (n = 19)	*P-*value
Sex(males/females)	11/9	9/10	0.752
Age (years)	28.00 ± 1.10	27.11 ± 1.16	0.581
NAR	0.36 ± 0.03	0.38 ± 0.03	0.685
NCV	15.11 ± 0.27	14.93 ± 0.24	0.627
nNO	272.5 ± 18.14	282.4 ± 17.61	0.709
SP	290.50 ± 36.89	315.80 ± 24.84	0.577
VIP	0.41 ± 0.04	0.40 ± 0.05	0.848
NPY	6.19 ± 1.13	5.65 ± 0.91	0.715

All data are presented as mean ± SEM.

AA = active acupuncture; SA = sham acupuncture; NAR = Total nasal airway resistance at 75 Pa (Pa·cm^−3^·s^−1^); NCV = Total nasal cavity volume from 0–5 cm in the nostril (cm^3^); nNO = exhaled nasal nitric oxide (ppb); SP = substance P (pg/ml); VIP = vasoactive intestinal peptide (ng/ml); NPY = neuropeptide Y (pg/ml). The differences in NAR, NCV, nNO, SP, VIP and NPY between two groups were assessed by analysis of variance (ANOVA) for repeated measures. The difference in age was compared between the groups by independent-samples t test. The difference in sex ratio between two groups was compared by Chi-squared tests *P < 0.05, **P < 0.01, ***P < 0.001.

**Table 2:** The change in the nasal ventilation, following acupuncture.

Time	AA group (n = 20)		SA group (n = 19)		*P-*value
	Better	Unchanged	Better	Unchanged	
30 minutes	19(95%)	1(5%)	2(10.5%)	17(89.5%)	<0.001***
2 hours	18(90%)	2(10%)	1(5.2%)	18(94.8%)	<0.001***
24 hours	16(80%)	4(20%)	0(0%)	19(100%)	<0.001***

Data are presented as N (%) values.

AA = active acupuncture; SA = sham acupuncture. Differences between the groups were assessed by Chi-Square tests; ***P < 0.001.

**Table 3:** The change in nasal patency, exhaled nasal nitric oxide, and neuropeptides, following acupuncture.

Characteristic	Time	AA group (n = 20)	SA group (n = 19)	*P-*value
NAR	Baseline	0.36 ± 0.03	0.38 ± 0.03	0.685
	30 mins	0.23 ± 0.01	0.33 ± 0.02	< 0.001***
	2 hours	0.24 ± 0.01	0.36 ± 0.02	< 0.001***
	24 hours	0.28 ± 0.02	0.35 ± 0.02	0.027*
NCV	Baseline	15.11 ± 0.27	14.93 ± 0.24	0.627
	30 mins	16.66 ± 0.45	14.81 ± 0.26	0.037*
	2 hours	16.47 ± 0.49	14.79 ± 0.28	0.006**
	24 hours	15.84 ± 0.33	14.93 ± 0.24	0.018*
nNO	Baseline	272.5 ± 18.14	282.4 ± 17.61	0.709
	30 mins	290.1 ± 32.91	272.5 ± 21.92	0.935
	2 hours	233.6 ± 16.49	275.0 ± 19.00	0.111
	24 hours	184.0 ± 10.98	276.4 ± 18.82	< 0.001***
SP	Baseline	290.5 ± 36.89	315.8 ± 24.84	0.577
	30 mins	511.5 ± 68.24	345.1 ± 29.89	0.037*
	2 hours	410.8 ± 83.86	357.1 ± 27.46	0.555
	24 hours	375.90 ± 35.23	343.2 ± 24.26	0.456
VIP	Baseline	0.41 ± 0.04	0.40 ± 0.05	0.848
	30 mins	0.47 ± 0.05	0.42 ± 0.05	0.581
	2 hours	0.49 ± 0.05	0.42 ± 0.05	0.357
	24 hours	0.46 ± 0.05	0.41 ± 0.03	0.465
NPY	Baseline	6.19 ± 1.13	5.65 ± 0.91	0.715
	30 mins	12.94 ± 2.56	6.64 ± 1.02	0.034*
	2 hours	12.91 ± 1.96	5.90 ± 0.84	0.003**
	24 hours	8.90 ± 2.12	5.86 ± 0.85	0.205

AA = active acupuncture; SA = sham acupuncture; NAR = Total nasal airway resistance at 75 Pa (Pa·cm^−3^·s^−1^); NCV = Total nasal cavity volume from 0–5 cm in the nostril (cm^3^); nNO = exhaled nasal nitric oxide (ppb); SP = substance P (pg/ml); VIP = vasoactive intestinal peptide (ng/ml); NPY = neuropeptide Y (pg/ml). All data are presented as mean ± SEM. The differences in NAR, NCV, nNO, SP, VIP and NPY between two groups were assessed by analysis of variance (ANOVA) for repeated measures; *P < 0.05, **P < 0.01, ***P < 0.001.
